# Acoine, a New Local Anesthetic

**Published:** 1899-12

**Authors:** 


### Acoine, a New Local Anesthetic.

R. L. Randolph (*Ophthalmic Record*, August; *Therapeutic Gazette*, October), refers to observations of Trolldeiner (*Therapeutische Monatshefte*, January), on the discovery of a new local anæsthetic, acoine. Acoine is a white powder, quite soluble in water in the proportion used in the author's experiments; *i. e.*, four grains and one-half to the ounce of water. It is derived from granine, which is found in almost all animal and vegetable cellular tissue. Acoine is related to caffeine and theobromine. Randolph concludes that:

1. Acoine in solutions of one in one hundred, and one in three hundred, produces satisfactory anæsthesia in an unirritated eye in about the same length of time as cocaine.

2. In more than one case where the eye was congested repeated instillations of acoine were inadequate to produce satisfactory anæsthesia.

3. Inspection of the cornea with a high-power lens failed to show any defects in the epithelium after its use.

4. Acoine has no effect upon accommodation.

5. It has no effect upon the size of the pupil.

6. It does not increase intraocular tension.

7. Several experiments showed that the *Staphylococcus pyogenes albus* did not grow in agar which contained acoine in the proportion used in the clinic, and furthermore, that exposure of this organism to the action of acoine for twenty-four hours was followed by its death. This would look as though acoine were not only an inhibitor of the growth of the *Staphylococcus albus*, but that it also killed this organism after a certain length of time. It is, of course, evident that conclusions drawn from this limited experience with acoine may have to undergo more or less modification with further trial.—*New York Medical Journal.*

